# Supplementary material for: Assessing the Viability of Segmental Aneuploid Embryos: A Chromosomal Concordance Study of 175 Human Blastocysts
Source: Int J Mol Sci. 2025 May 30;26(11):5284. doi: 10.3390/ijms26115284 (PMC12155557; doi:10.3390/ijms26115284)
Supplement: Supplementary file 1 [file ijms-26-05284-s001.zip › ijms-3568232-supplementary.pdf]

Supplement Table S1. The list of NGS results segmental mosaicism of original biopsy and inner cell mass by PGT-A

N0.	:mbryo quality	Seg-M of original TE biopsy	Classification of PGT-A of ICM	NGS results of ICM
1	5BC	Mosaicism, XX. (30% mosaic gain of 1p36.22~36.12, 2p24.1~11.1, 12q15~23.1; 30% mosaic loss of 13q33.1~34)	EU	Euploidy, XX.
2	4AB	Mosaicism, XX. (70% mosaic loss of 1p36.33~36.22; 30% mosaic gain of 1p22.2~21.1(29%), 4p16.1~q12(26%); 30% mosaic loss of 7q21.11~21.3, 10q22.3~23.31)	EU	Euploidy, XX.
3	4BB	Mosaicism, XY. (60% mosaic loss of 2p25.3~13.2; 40% mosaic loss of 6q12~14.3)	EU	Euploidy, XY.
4	4BB	Mosaicism, XX. (60% mosaic loss of 2q31.1~37.3; 40% mosaic gain of 11p11.2~q13.4 & 13q13.3~14.2; 40% mosaic loss of 2q22.1~31.1 & 2p25.3~16.3 & 11q23.3~25 & Xp22.33~21.2; 30% mosaic gain of 1p36.23~q25.3 & 4p16.1~q23 & 5p15.1~q14.1 & 12p13.33~q13.2; 30% mosaic loss of 3q26.1~29 & 6p25.3~12.3 & 6q14.1~27 & 13q22.3~34 & 14q24.3~32.33 & 16p11.2~q24.3 & Xq25~28)	EU	Euploidy, XX.

5	5BB	Mosaicism, XY. (50% mosaic loss of 2p25.3~14; 30% mosaic gain of 2q24.2~31.1)	EU	Euploidy, XY.
6	5BB	Mosaicism, XX. (30% mosaic loss of 2p25.3~25.1, 5p15.33~15.2, Xq27.1~28, Xq21.1~22.3)	EU	Euploidy, XX.
7	4BB	Mosaicism, XX. (50% mosaic loss of 4q13.1~4q34.3; 40% mosaic loss of Xp22.33~11.4 & Xq13.3~28; 30% mosaic gain of 1p32.3~21.1; 30% mosaic loss of 18p11.32~q21.1)	EU	Euploidy, XX.
8	4BB	Mosaicism, XX. (30% mosaic gain of 5p14.1~q15, 11p12~q12.2; 30% mosaic loss of Xq21.1~28)	EU	Euploidy, XX.
9	5AB	Mosaicism, XY. (30% mosaic gain of 5p12~q11.2, 6q22.31~23.2; 30% mosaic loss of 13q31.1~31.3)	EU	Euploidy, XY.
10	5AB	Mosaicism, XX. (40% mosaic gain of 5p13.2~q14.3; 30% mosaic loss of 5p15.33~15.2, 8p23.3~11.22, 13q33.1~34)	EU	Euploidy, XX.
11	5AA	Mosaicism, XX. (40% mosaic loss of 5p15.33~15.2, 11q24.1~25; 30% mosaic loss of 14q32.12~32.33, Xq27.3~28)	EU	Euploidy, XX.

12	5BA	Mosaicism, XX. (70% mosaic loss of 7p22.3~14.3; 50% mosaic loss of 7q35~36.3; 30% mosaic loss of 7p14.2~q35; 30% mosaic gain of 5p15.1~5p13.2, 5q12.1~5q14.3, 5q31.2~5q33.2, chr17 & chr19 & 20p13~q13.12)	EU	Euploidy, XX.
13	5BB	Mosaicism, XY. (70% mosaic loss of 7p22.3~11.2; 40% mosaic loss of 6q25.3~27; 30% mosaic gain of 20p12.3~11.23)	EU	Euploidy, XY.
14	5BB	Mosaicism, XX. (30% mosaic gain of 7q21.13~22.3; 30% mosaic loss of 7p22.3~11.2, 5p15.33~15.2; 20% mosaic loss of 2p25.3~25.1)	EU	Euploidy, XX.
15	5BB	Mosaicism, XX. (50% mosaic gain of 7q31.1~36.3)	EU	Euploidy, XX.
16	5AA	Mosaicism, XX. (50% mosaic loss of 7q35~36.3; 30% mosaic loss of 5p15.33~14.3, 11q24.1~25, 14q32.13~32.33, Xq27.1~28)	EU	Euploidy, XX.
17	4BB	Mosaicism, XX. (60% mosaic gain of 8q11.23~24.3; 50% mosaic loss of Xq21.1~21.33; 40% mosaic loss of 2p25.3~24.1)	EU	Euploidy, XX.
18	4BC	Mosaicism, XX. (60% mosaic loss of 8p23.3~11.21 & 10q23.1~26.3; 40% mosaic gain	EU	Euploidy, XX.

		of 6p25.3~21.2; 40% mosaic loss of 8p11.21~q24.3)		
19	4BA	Mosaicism, XY. (50% mosaic loss of 9q33.2~34.3)	EU	Euploidy, XY.
20	5BB	Mosaicism, XX. (30% mosaic gain of 9q12~21.2)	EU	Euploidy, XX.
21	4BB	Mosaicism, XX. (80% mosaic gain of 9q22.1~34.3)	EU	Euploidy, XX.
22	5BC	Mosaicism, XX (30% mosaic loss of 11q24.2~25)	EU	Euploidy, XX.
23	5BB	Mosaicism, XX. (40% mosaic loss of 11q22.3~25; 30% mosaic gain of 14q23.3~24.3)	EU	Euploidy, XX.
24	5BB	Mosaicism, XY. (30% mosaic loss of 11q24.2~25)	EU	Euploidy, XY.
25	5BB	Mosaicism, XY. (80% mosaic loss of 12q14.3~24.33)	EU	Euploidy, XY.
26	4BB	Mosaicism, XX. (30% mosaic gain of 12p12.3~11.21 & 17p11.2~q21.32)	EU	Euploidy, XX.
27	5BB	Mosaicism, XX. (30% mosaic loss of 14q32.13~32.33(29%))	EU	Euploidy, XX.

28	5BB	Mosaicism, XX. (50% mosaic loss of 14q32.12~32.33; 40% mosaic loss of 5p15.33~15.2, 6q26~27, Xq27.1~28; 30% mosaic loss of 1q42.13~44, 11q23.3~25)	EU	Euploidy, XX.
29	5BA	Mosaicism, XX. (30% mosaic loss of 14q32.12~32.33)	EU	Euploidy, XX.
30	5BB	Mosaicism, XX. (50% mosaic loss of 17p13.3~11.1; 30% mosaic gain of 17q11.2~25.3; 30% mosaic loss of 2p25.3~24.1, 7q34~36.3)	EU	Euploidy, XX.
31	6BB	Mosaicism, XX. (40% mosaic gain of 21p11.1~q21.3, Xq22.3~23; 30% mosaic gain of 5p15.1~q12.2, 14q11.2~22.1; 30% mosaic loss of 2p25.3~22.3, 4q28.3~35.2)	EU	Euploidy, XX.
32	4CB	Mosaicism, XX.(50% mosaic loss of 7p22.3~7p11.2)	EU	Euploidy, XX.
33	4BC	Mosaicism, XX. (30% mosaic loss of Xq12~21.33)	EU	Euploidy, XX.
34	5BB	Mosaicism, XX. (40% mosaic loss of Xq27.2~28)	EU	Euploidy, XX.
35	5BB	Mosaicism, XX. (30% mosaic loss of Xq27.3~28)	EU	Euploidy, XX.

36	5AB	Mosaicism, XX. (40% mosaic loss of Xq13.1~21.33; 30% mosaic gain of Xp22.33~22.11; 30% mosaic loss of 21p11.1~q21.2)	EU	Euploidy, XX.
37	5BA	Mosaicism, XX. (50% mosaic gain of Xp22.33~11.22)	EU	Euploidy, XX.
38	5BB	Mosaicism, XX. (30% mosaic loss of Xq11.2~22.3)	EU	Euploidy, XX.
39	4BB	Mosaicism, XX. (40% mosaic loss of 1q41~44)	Seg-M	Mosaicism, XX. (30% mosaic loss of 14q32.2~32.33)
40	5AB	Mosaicism, XX. (30% mosaic loss of 2p25.3~24.1, 7q35~36.3, 11q24.2~25)	Seg-M	Mosaicism, XX. (50% mosaic loss of 7q11.21~36.3)

41	4BB	Mosaicism, XY. (40% mosaic gain of chr3 & 14q11.2~24.1 & 16p13.3~11.2; 40% mosaic loss of 1q32.2~44, 4q34.3~35.2, 12q24.21~24.33, 13q31.1~34, 17q23.2~25.3; 30% mosaic gain of chr3 8p23.3~q24.12; 30% mosaic loss of 1p36.22~35.2, 1q23.3~31.1, 6p24.2~22.3, 6q25.3~27, 6q13~15)	Seg-M	Mosaicism, XY.(50% mosaic loss of 15q11.2~22.2; 40% mosaic gain of 3q25.2~29 & 6q14.3~25.3 & 10q24.2~26.11; 40% mosaic loss of 8p23.3~21.2 & 16p13.3~q12.2 & chr21; 30% mosaic gain of 1q21.3~44 & 2q22.1~37.1 & 8q22.2~24.23; 30% mosaic loss of 12p13.33~11.1 & chr20)
42	5BB	Mosaicism, XY. (80% mosaic loss of 5q13.2~35.3; 40% mosaic loss of 1q23.3~44)	Seg-M	Mosaicism, XY. (30% mosaic loss of 5q13.2~35.3)

43	5AB	Mosaicism, XX. (40% mosaic loss of 5p15.33~15.2, 11q24.2~25)	Seg-M	Mosaicism, XX. (40% mosaic loss of 21q22.12~22.3; 40% mosaic loss of 3p22.1~14.1, 5p15.33~15.2, 8p23.3~22; 30% mosaic gain of 1p36.13~21.2, 1q25.3~42.13; 30% mosaic loss of 2p25.3~24.1, Xp22.13~11.3)
44	5BB	Mosaicism, XX. (50% mosaic loss of 5q31.1~35.3; 30% mosaic gain of 1p31.1~21.3(27%); 30% mosaic loss of 17q21.31~23.2, 18q12.1~23, Xq21.1~21.33)	Seg-M	Mosaicism, XX. (60% mosaic loss of 5q31.1~35.3)
45	4BB	Mosaicism, XX. (40% mosaic loss of 5p15.33~15.2, 7q35~36.3; 30% mosaic loss of 10q25.1~26.3, 11q24.2~25, Xq13.3~22.2)	Seg-M	Mosaicism, XX. (30% mosaic gain of 19q13.32~13.43; 30% mosaic loss of 10q25.3~26.3, 13q32.1~34)

46	5BC	Mosaicism, XX. (70% mosaic gain of 5p13.3~q14.3; 40% mosaic gain of 5p15.33~13.3, 5q14.3~35.3; 40% mosaic loss of Xq27.1~28; 30% mosaic gain of 19q13.11~13.41; 30% mosaic loss of Xq21.1~22.1)	Seg-M	Mosaicism, XX. (30% mosaic gain of 5p15.31~q35.2, 8q12.1~24.22)
47	5BC	Mosaicism, XY. (80% mosaic loss of 6q12~27; 30% mosaic loss of 8q23.1~24.3, 9p21.3~13.3, chrY)	Seg-M	Mosaicism, XY. (60% mosaic loss of 6q12~27)
48	5BB	Mosaicism, XY. (70% mosaic loss of 7p22.3~q22.1; 30% mosaic loss of 7q22.1~36.3, 14q32.12~32.33)	Seg-M	Mosaicism, XY. (40% mosaic loss of 7p22.3~q22.1; 30% mosaic gain of chr2; 30% mosaic loss of 7q22.1~36.3)
49	5BB	Mosaicism, XX. (30% mosaic loss of 7q31.1~31.33)	Seg-M	Mosaicism, XX. (30% mosaic loss of 11q24.2~25, Xq27.2~28)
50	5BB	Mosaicism, XX. (30% mosaic loss of Xq12~21.33)	Seg-M	Mosaicism, XX. (30% mosaic gain of 11p11.2~q12.3)

51	6BB	Mosaicism, XX. (50% mosaic loss of 13q31.1~34)	Seg-M	Mosaicism, XX. (30% mosaic gain of 5q32~33.3; 30% mosaic loss of Xq27.3~28)
52	5CB	Mosaicism, XX. (40% mosaic loss of 16q12.2~24.3; 30% mosaic gain of chrX)	Seg-M	Mosaicism, XX. (40% mosaic loss of chr3 & 5p15.33~15.1; 30% mosaic gain of chr6 & 16q13~24.3)
53	5BB	Mosaicism, XX. (50% mosaic loss of 17q24.2~25.3)	Seg-M	Mosaicism, XX. (30% mosaic loss of 17q24.3~25.3)
54	4BB	Mosaicism, XY. (50% mosaic loss of 22q13.1~13.33)	Seg-M	Mosaicism, XY. (70% mosaic loss of 22q13.1~13.33)
55	5BB	Mosaicism, XX. (30% mosaic gain of 22q11.1~13.31; 30% mosaic loss of chr7, chr9, chr11)	Seg-M	Mosaicism, XX. (30% mosaic loss of 2p25.3~25.1)
56	4BB	Mosaicism, XX. (30% mosaic loss of Xq21.1~21.33, Xq27.3~28)	Seg-M	Mosaicism, XX. (30% mosaic gain of 10q24.2~26.3)
57	5AB	Mosaicism, XX. (30% mosaic loss of Xq12~21.31)	Seg-M	Mosaicism, XX. (30% mosaic gain of 9q21.11~34.3)

58	4BB	Mosaicism, XX. (30% mosaic loss of Xq11.1~28)	Seg-M	Mosaicism, XX. (30% mosaic gain of 8q23.3~8q24.22)
59	4BB	Mosaicism, XX. (50% mosaic gain of 3p14.3~q13.31; 40% mosaic loss of 4p15.1~13, 17q11.2~23.3; 30% mosaic loss of chr7, chr11, chr13)	Who-M	Mosaicism, XX.(40% mosaic loss of chr10, chr20)
60	5BB	Mosaicism, XX. (60% mosaic gain of 5p15.1~13.3; 30% mosaic loss of 11q24.2~25)	Who-A	Aneuploidy, XX. +18.
